# Novel and Haplotype Specific MicroRNAs Encoded by the Major Histocompatibility Complex

**DOI:** 10.1038/s41598-018-19427-6

**Published:** 2018-03-01

**Authors:** P. M. Clark, N. Chitnis, M. Shieh, M. Kamoun, F. B. Johnson, D. Monos

**Affiliations:** 1 Department of Pathology and Laboratory Medicine, The Children’s Hospital of Philadelphia, Philadelphia, PA 19104 USA; 2 Department of Pathology and Laboratory Medicine, Perelman School of Medicine, University of Pennsylvania, Philadelphia, PA 19104 USA

## Abstract

The MHC is recognized for its importance in human health and disease. However, many disease-associated variants throughout the region remain of unknown significance, residing predominantly within non-coding regions of the MHC. The characterization of non-coding RNA transcripts throughout the MHC is thus central to understanding the genetic contribution of these variants. Therefore, we characterize novel miRNA transcripts throughout the MHC by performing deep RNA sequencing of two B lymphoblastoid cell lines with completely characterized MHC haplotypes. Our analysis identifies 89 novel miRNA transcripts, 48 of which undergo Dicer-dependent biogenesis and are loaded onto the Argonaute silencing complex. Several of the identified mature miRNA and pre-miRNA transcripts are unique to specific MHC haplotypes and overlap common SNPs. Furthermore, 43 of the 89 identified novel miRNA transcripts lie within linkage disequilibrium blocks that contain a disease-associated SNP. These disease associated SNPs are associated with 65 unique disease phenotypes, suggesting that these transcripts may play a role in the etiology of numerous diseases associated with the MHC. Additional *in silico* analysis reveals the potential for thousands of putative pre-miRNA encoding loci within the MHC that may be expressed by different cell types and at different developmental stages.

## Introduction

The major histocompatibility complex (MHC) is a ~4 Mbp stretch of the human genome located on the short arm of chromosome 6, which encompasses numerous genes involved in a variety of immunological processes. It is amongst the most gene dense and variable regions of the human genome^1–3^, and has been shown to harbor the highest density of disease associated variants of any 4Mbp stretch throughout the human genome^4^. However, despite the numerous disease associations throughout the MHC, the functional contribution of these variants remains unclear, due in part to the complex linkage disequilibrium (LD) pattern within the MHC and the numerous SNPs that lie within non-coding regions of the MHC. Although elucidating the functional significance of these SNPs remains a significant challenge, recent research suggests that ~90% of causal autoimmune disease variants reside within non-coding regions of the human genome, with ~60% mapping to immune cell enhancer-like elements which gain histone acetylation following immune stimulation, hence contributing to the transcription of non-coding RNAs^5^. Consequently, the identification and characterization of functional non-coding transcripts within the MHC is an essential step towards elucidating the contribution of non-coding variants within the MHC to human health and disease.

The MHC has been reported to encode 12 annotated precursor miRNA hairpin (pre-miRNA) loci (miRBase release 21)^6^. MicroRNAs (miRNAs) are a class of single stranded, non-coding RNA (ncRNA) transcripts, approximately 22 nucleotides in length that attenuate the translation of targeted mRNA transcripts^7^ following loading of the miRNA transcript onto the Argonaute (Ago) RNA induced silencing complex (RISC)^8^. Recent research to characterize the miRNA transcriptome of various tissues has led to the discovery of numerous novel miRNA transcripts^9–15^, greatly expanding upon the currently annotated set of 2,813 mature miRNA transcripts (miRBase database release 21)^6^. One miRNA in particular that was born out of this effort, miR-6891-5p^11^, has been shown to originate from within a highly conserved intronic segment of the HLA-B gene. This miRNA we recently reported to regulate the expression of numerous immunologically related transcripts, including those encoding the heavy chain of IgA^16^. These findings raise the possibility that additional miRNA transcripts that originate from within polymorphic regions of the MHC including other HLA genes, may play a significant role in regulating a number of biological processes.

Despite the discovery of thousands of novel miRNA transcripts located throughout the genome, the identification and quantification of miRNA transcripts originating from within polymorphic loci such as the MHC remains a significant challenge due to inherent sequence differences between the reference genome and the transcripts originating from any given individual. These differences are particularly problematic when mapping miRNA transcripts, since most miRNA mapping pipelines allow for no more than one mismatch between a sequenced read and the reference genome. Although stringent read mapping parameters are necessary to reduce the number of spuriously mapped reads, such an approach is inherently unable to align short reads that differ from the locus of origin within the reference genome by more than one base. Therefore, in order to identify and better characterize the miRNA transcripts originating from within the polymorphic MHC, we performed deep RNA sequencing of miRNA transcripts obtained from two B lymphoblastoid cell lines (BLCLs), with completely characterized, homozygous MHC haplotypes.

Our approach facilitates the accurate mapping of short RNA-seq reads derived from the miRNA transcripts of two MHC homozygous BLCLs (PGF and COX) to their respective reference MHC haplotype sequence^1,2^. Analysis of the mapped reads obtained from these two cell lines reveals the existence of numerous novel miRNA transcripts originating from within the MHC including several that are only present within specific MHC haplotypes. Nearly half of the identified novel miRNA transcripts (n = 89) are located within a LD block that also contains a disease associated SNP, suggesting that these novel miRNA transcripts may play a role in the etiology of the numerous diseases associated with the MHC. Additional, *in silico* analysis of the two MHC haplotype sequences (PGF and COX) reveals the potential for thousands of additional putative pre-miRNA encoding loci throughout the MHC, which may be expressed in various tissues, phenotypes and developmental states.

## Results

### Identifying Novel miRNA Transcripts of the MHC

**Figure 1:**
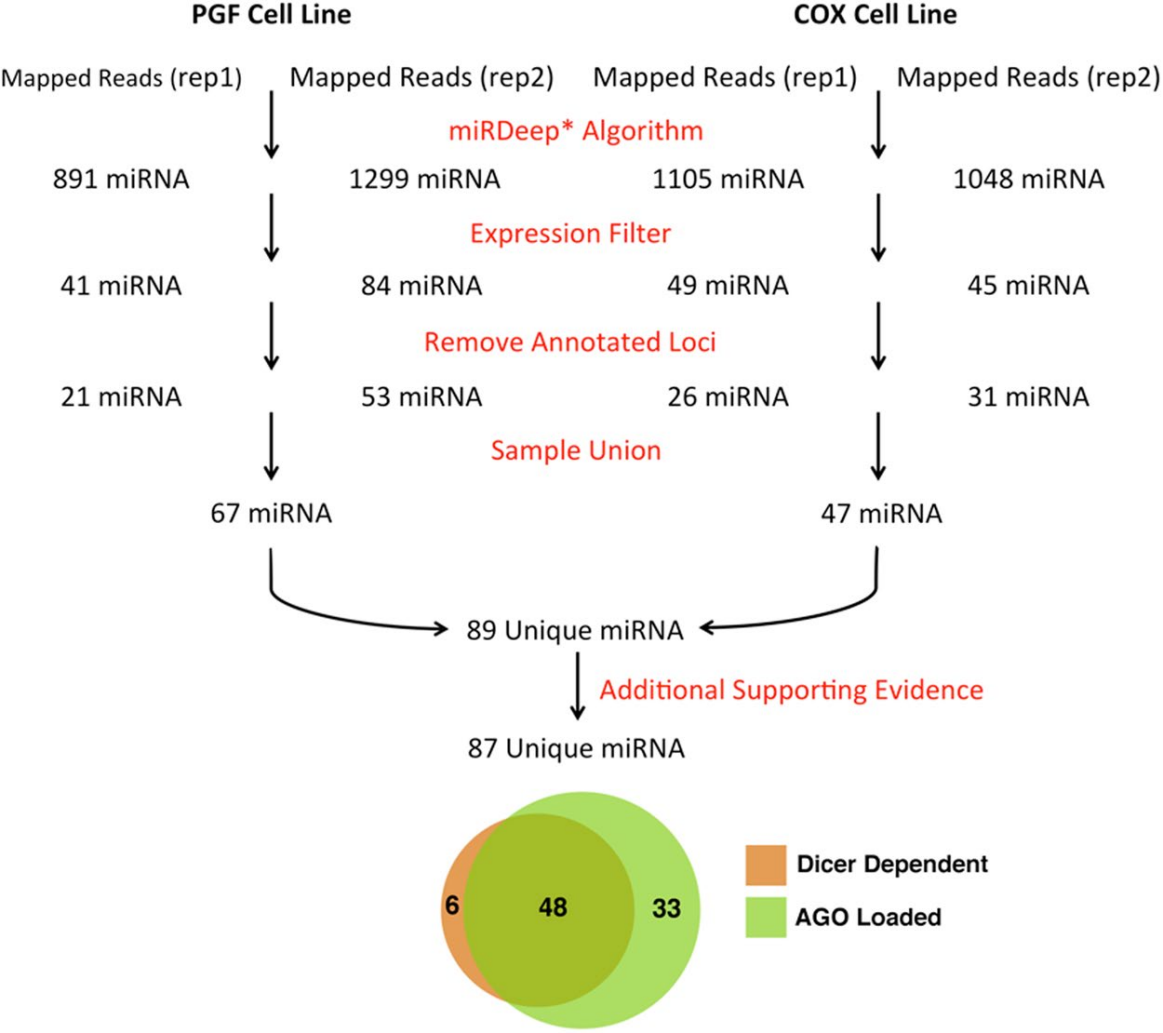
Computational pipeline to discover novel miRNAs expressed within two lymphoblastoid cell lines (PGF and COX). RNA-Seq was performed on two biological replicates of two homozygous BLCLs with completely characterized MHC haplotypes, PGF and COX. Mapped reads were utilized to discover significantly expressed novel miRNAs from each RNA-seq run using miRDeep*. In total 89 unique miRNAs were discovered from all four datasets, with 87 of them having additional functional evidence (either loaded onto the Argonaute silencing complex or are formed in a Dicer dependent manner).

Deep sequencing of the miRNA transcriptome was performed on two BLCLs, PGF and COX. These two cell lines were chosen because they are homozygous for the MHC region and both have distinct and completely sequenced MHC haplotypes, facilitating unambiguous mapping of short RNA-seq reads to polymorphic regions throughout the MHC. Although the genome-wide transcriptional profile of BLCLs has been previously reported^9,12^, these efforts do not adequately describe the profile of haplotype and allele specific miRNA transcripts originating from within the polymorphic MHC. For this reason, our experimental design and analysis pipeline were developed, facilitating the identification and characterization of novel miRNA transcripts within the MHC. A modified version of a previously established analytical pipeline^9^ was utilized for the discovery of novel miRNAs encoded within the MHC using mapped RNA-seq reads from the two biological replicates of each cell line (Fig. 1).

Two biological replicates of each cell line were sequenced, generating a total of four RNA-seq datasets. The reads from each sample were mapped to the reference genome (HG38) that had been modified to include either the PGF or COX MHC haplotype reference sequence, depending on the cell line of origin. Approximately 90% of the raw reads generated by each sequencing run were aligned to their respective haplotype specific reference genome. The set of aligned reads was subsequently used for the identification of novel miRNAs transcripts using the miRDeep* algorithm^17^. Only the identified, novel miRNAs that were significantly expressed within each individual sequencing run and did not overlap with an annotated miRNAs (mirBase release 21) or exonic, protein coding sequence were retained for further analysis. In total, 89 novel mature miRNAs were identified from the analysis of RNA-Seq data obtained from the two cell lines. The majority (82%) of identified novel miRNAs lie within intergenic regions of the MHC, with 16 identified novel mature miRNAs residing within the introns of 14 unique genes; *ATF6B*, *C2*, *CSNK2B*, *DDX39B*, *GABBR1*, *HGC20*, *HLA-DRB5*, *LY6G6C*, *MSH5*, *NFKBIL1*, *NOTCH4*, *SLC39A7*, *TNXB*, and *TRIM31*.

### Supporting Functional Evidence for Identified Novel miRNAs

Since the majority of mature miRNA transcripts are formed through the canonical Dicer-dependent biogenesis pathway^18–20^, Dicer knockdown or silencing experiments have been used to identify mature miRNAs that are formed in a Dicer-dependent manner^21^. Our Dicer silencing experiment demonstrates that the expression of 54/89 (60%) of the identified novel miRNAs are significantly attenuated following Dicer silencing (pval ≤ 0.05) as evaluated by qPCR, indicating that the biogenesis of at least these 54 mature miRNAs is Dicer dependent.

Mature miRNAs facilitate the suppression of targeted mRNA transcripts following miRNA loading onto the Ago RISC^8^. For this reason, Ago CLIP-seq datasets, in which the Ago protein is immunoprecipitated and the bound RNA fraction sequenced, have been previously used to identify functional miRNA targets and validate functional novel miRNAs^9,10^. We performed a meta-analysis on 41 Ago CLIP-seq datasets from 4 independent studies^22–25^ in order to provide evidence that our 89 novel miRNA transcripts are functional miRNAs that are loaded onto the Ago silencing complex. Our results indicate that 81 of the 89 (91%) identified novel miRNAs described by this study are found to be loaded onto the Ago silencing complex. These results along with the Dicer silencing results demonstrate that 48/89 of the novel miRNAs were found to be formed in a Dicer dependent manner and also loaded onto the Ago silencing complex. The genomic locus of origin and supporting evidence (Ago support and Dicer dependency) for each identified novel mature miRNA is provided in Supplemental Table 1.

### Haplotype Conservation of Novel miRNAs

**Figure 2:**
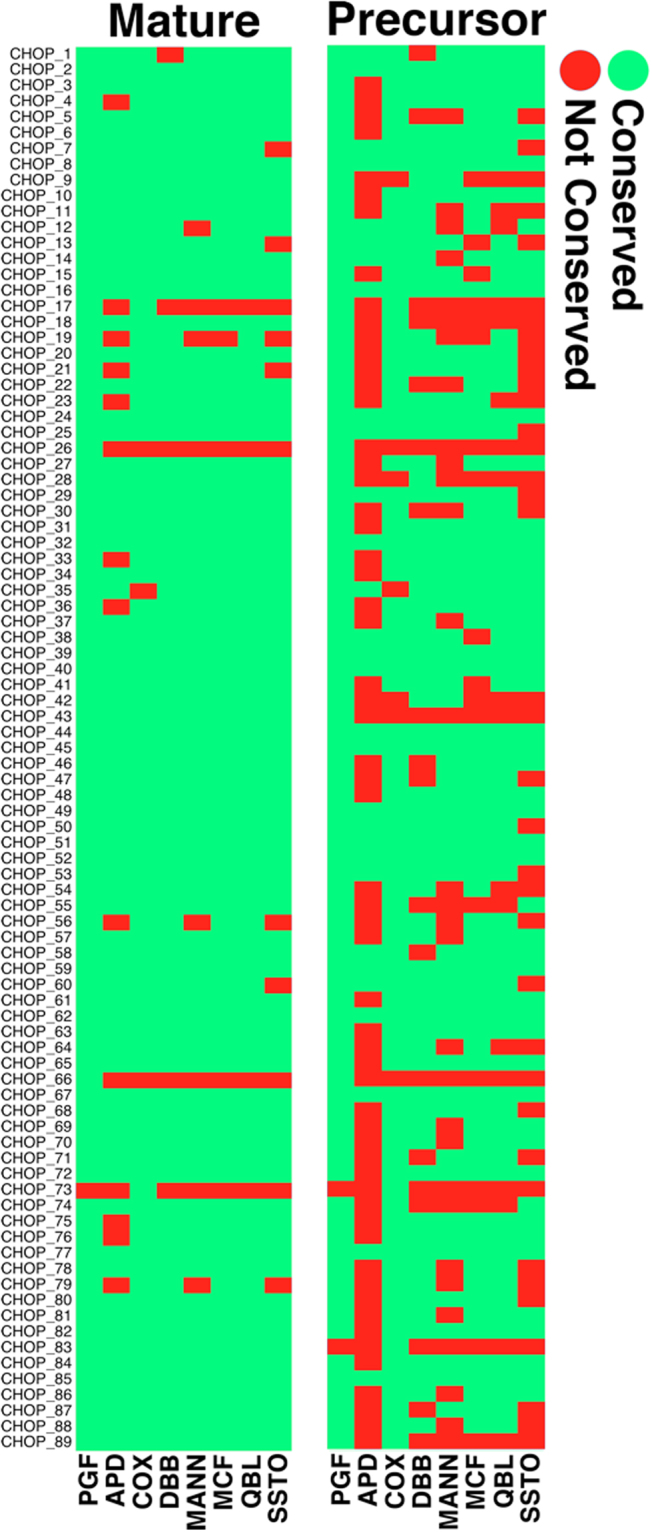
Sequence conservation of identified novel mature and pre-miRNA hairpin sequences across all known MHC haplotypes.

In order to determine the presence of each identified novel mature and pre-miRNA encoding sequence within the set of annotated complete (PGF and COX) as well as partially complete MHC haplotypes (MCF, DBB, MANN, APD, SSTO and QBL), each miRNA sequence was compared with each annotated MHC haplotype sequence using BLAST. Only perfectly matched sequences (100% sequence identity between the query sequence and the reference MHC haplotype) were considered to be conserved within a particular MHC haplotype (Fig. 2). Our results indicate that while the majority of mature miRNAs are conserved amongst the set of eight annotated MHC haplotypes analyzed, a subset of mature miRNAs are found to exist within specific MHC haplotypes (Fig. 2). Of note CHOP_66 lies within intron 5 of HLA-DRB5 and is composed of reads that map uniquely to this locus. It was also observed that the identified pre-miRNA sequences are less conserved across the analyzed haplotypes as compared to the mature miRNA sequences (Fig. 2).

It should also be noted that the presence of the 89 miRNA sequences in the PGF and COX MHC sequences determined computationally (Fig. 2) is distinct from the presence of the same miRNAs as actually expressed in PGF and COX cells and confirmed experimentally. The mere presence of the miRNA sequences in the MHC of either cell does not necessitate their expression.

### Sequence Homology of Novel miRNAs to Known miRNAs

**Figure 3:**
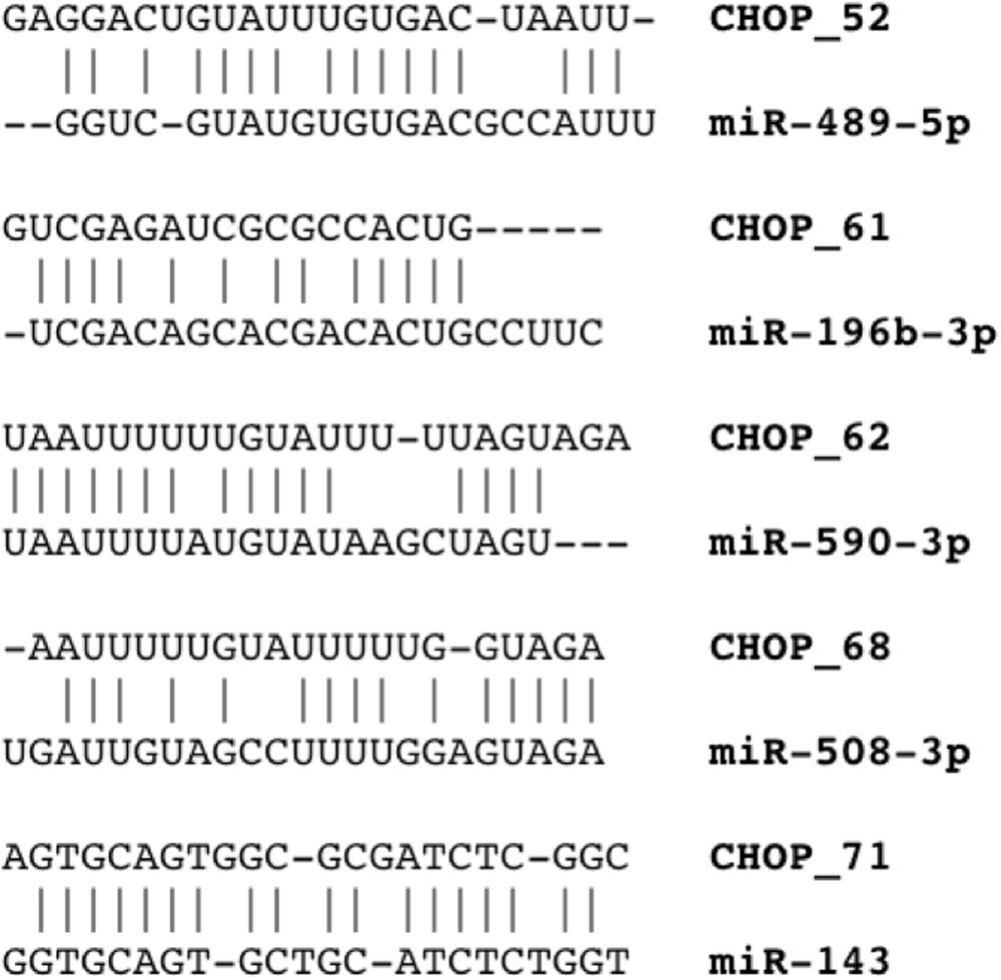
Sequence homology between newly identified miRNAs and previously identified oncomiRs.

The set of identified 89 novel mature miRNAs were compared against all known, previously annotated miRNAs (miRBase release 21) in order to identify closely related miRNAs that share partial sequence homology, which may be indicative of a shared target repertoire and physiological function. For this purpose, each identified novel miRNA was aligned pairwise with every annotated mature miRNA sequences (miRBase release 21). Several identified novel miRNAs closely matched the sequences of annotated miRNAs of known physiological function that have been demonstrated to play a role in oncogenesis (Fig. 3), including miR-489^26^, miR-196^27,28^, miR-590^29^, miR-508^30^ and miR-143^31^.

### *In Silico* Discovery of Putative Pre-miRNA Encoding Loci

**Figure 4:**
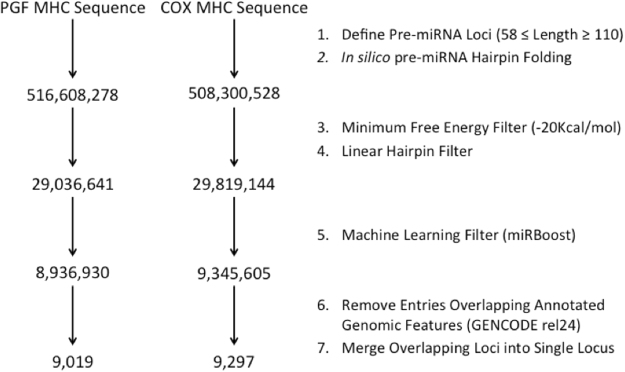
Computational prediction pipeline and putative miRNA loci identified from the annotated MHC haplotype sequences of PGF and COX lymphoblastoid cell lines.

Many recently discovered miRNAs, including those described within our current work, rely on the identification of novel miRNA from RNA-seq data derived from a variety of tissue types. However, such an approach is inherently limited to discovering only the set of expressed miRNA transcribed within the interrogated tissue types. Given the demonstrated tissue specific RNA expression patterns and limited number of cell lines with completely characterized MHC haplotypes, we have developed a computational pipeline to identify all putative pre-miRNA encoding loci within the reference MHC haplotype sequences of both PGF and COX that may be expressed by various cell types and developmental stages. The developed pipeline is a multi-step process designed to exhaustively interrogate the propensity of genomic loci to form stable, pre-miRNA hairpin structures (Fig. 4). Our analysis identified 9,019 and 9,297 loci containing at least one pre-miRNA hairpin structure for PGF and COX MHC haplotypes respectively. The sequences of 4,487 of the putative pre-miRNA encoding loci identified within the PGF MHC haplotype are 100% conserved within the COX MHC haplotype (4,487/9,019, ~50%). Overall, 11 of the 12 annotated pre-miRNAs (miRbase release 21) located within the MHC were also identified by our computational analysis pipeline. One miRNA, hsa-miR-6833 was filtered out because the pre-miRNA hairpin was found to have a minimum free energy (MFE) of −19.1Kcal/mol, which is greater (less energetically favorable) than the cutoff threshold of −20Kcal/mol. Furthermore 80 of the 89 (90%) pre-miRNAs identified through the analysis of RNA-Seq datasets, were also identified through our *ab initio* computational pre-miRNA annotation pipeline.

### Novel miRNA Loci within LD of Disease Associated SNPs

Although elucidating the physiological function of each identified novel miRNA is beyond the scope of this work, we seek to provide some insights into the potential role of the identified novel miRNA transcripts in the context of the many diseases reported to be associated with the MHC. Utilizing the wealth of annotated disease associated variants from GWAS studies^32,33^, we identified the subset of the miRNAs that lie within LD blocks of annotated disease associated variants within the MHC. Our results indicate that 43 of the 89, (~48%) identified novel miRNA transcripts from the analysis of RNA-seq datasets are within LD blocks containing 87 unique disease associated SNPs. These 87 SNPs have been associated with 65 unique phenotypes (Supplemental Table 3). In addition, 6,690 computationally derived putative pre-miRNA encoding loci identified from the analysis of the PGF MHC haplotype sequence were found to be in LD with 325 unique disease associated SNPs (data not shown).

## Discussion

Deep RNA sequencing of the miRNA transcriptome from two BLCLs with completely characterized MHC haplotypes (PGF and COX) has enabled the accurate alignment of short RNA-seq reads to each respective MHC haplotype sequence, facilitating the identification of 89 novel miRNA transcripts originating from within the polymorphic MHC region (Supplemental Table 1). Additional experimental validation of the set of identified novel miRNA transcripts demonstrates that 54/89 (~60%) of the identified novel miRNA transcripts are significantly attenuated (p ≤ 0.05) following Dicer silencing and that 81/89 (~91%) are loaded onto the Ago silencing complex. Together these data demonstrate that 54% (48/89) of the identified novel miRNAs are functional miRNAs that are formed through the canonical, Dicer-dependent biogenesis pathway^21^ and are loaded onto the Ago silencing complex. The number of identified novel miRNAs that undergo Dicer dependent biogenesis may however be an underestimate, since previous research demonstrates that functional mature miRNA transcripts can be formed independently of the Dicer enzyme^34–36^. Alternatively, the lower than expected number of identified miRNAs that undergo Dicer dependent biogenesis may be attributed to 1) accumulation of mature miRNA transcripts prior to Dicer silencing, 2) differential transcription of pre-miRNA hairpin transcripts amongst the two biological replicates or 3) differential Dicer processing of pre-miRNA hairpins^14,19^, resulting in the expression of miRNA isoforms (isomiRs) that may not be an optimal qPCR substrate for amplification using our designed set of primers.

The majority of identified miRNAs (73/89) originate from within the intergenic regions of the MHC, while 16/89 are located within an intron of an annotated host gene. We also find that 12/73 identified intergenic miRNAs are antisense to an annotated protein-coding gene. The mature miRNAs that lie within an intron, “mirtrons”, are formed following splicing of the mRNA transcript and have been shown to exist throughout the human genome^11,37,38^. Our previous research demonstrates that one such mirtron, miR-6891-5p, which is encoded within intron 4 of the HLA-B gene, plays an important physiological role by regulating the post transcriptional expression of nearly 200 mRNA transcripts that are involved in a variety of metabolic and immunological processes^16^. Interestingly, our current work suggests that *HLA-DRB5* also harbors an observed mirtron transcript, located within intron 5 of the *HLA-DRB5* gene. In addition, we find numerous pre-miRNA hairpins located within other HLA genes as predicted by our *in silico* analysis. Together these data suggest a novel, secondary function for select HLA transcripts, mediated by their encoded miRNAs.

The existence of MHC encoded miRNAs raises many questions related to the existence and prevalence of haplotype specific miRNAs. Although the majority of identified mature miRNA sequences are conserved across all eight known MHC haplotypes, several mature miRNA and pre-miRNA hairpin sequences are found to be unique to specific MHC haplotypes (Fig. 2). It is however important to note that only the MHC haplotypes of PGF and COX have been fully characterized, potentially resulting in a miRNA sequence being absent within a particular MHC haplotype simply because it lies within an uncharacterized region of that particular haplotype. For this reason, mature miRNAs were also intersected with common SNPs (dbSNP build 149), revealing that 27/89 (minor allele frequency – MAF ≥ 0.01) and 16/89 (MAF ≥ 0.05) of the identified novel mature miRNA transcripts contain at least one common SNP and are thus likely to be polymorphic across the population. Although the majority of identified novel mature miRNA sequences are conserved across most MHC haplotypes, the pre-miRNA hairpin sequences are far less conserved across MHC haplotypes. Polymorphisms within the pre-miRNA transcripts can influence the free energy and conformation of the secondary structure of the pre-miRNA hairpin structure, thereby influencing pre-miRNA processing by Dicer, which may result in a variety of expressed isomiRs^14,39,40^. Furthermore, an assessment of the haplotype conservation amongst the set of identified computationally predicted putative pre-miRNA encoding loci, reveals that ~50% (4,487/9,019) of these sequences are 100% conserved between the PGF and COX MHC haplotypes. Together these data suggest a diverse miRNA transcriptome, with the potential for a variety of isomiR transcripts that are defined by inherent differences amongst MHC haplotypes. It is anticipated that each of these isomiRs has a distinct target repertoire governed by the sequence specific interaction of the mature miRNA with targeted mRNA transcripts^14,41^.

The availability of an accurate, reference genome sequence is critical to ensure accurate mapping of short RNA-seq reads for the identification and quantification of novel miRNAs and isomiRs that are transcribed from within highly polymorphic loci such as the MHC. Because there are only two complete MHC haplotype sequences currently available (PGF and COX), our study is limited to the identification and quantification of novel miRNAs expressed by these two cell lines. However, because miRNA and isomiRs are expressed in a tissue and phenotype specific manner^41–43^, we sought to identify every potential miRNA encoding locus throughout the MHC using only the DNA reference haplotype sequences of PGF and COX as a guide. Our computational pipeline has identified thousands of potential pre-miRNA encoding loci throughout the MHC and serves as an atlas for the future identification and quantification of MHC encoded miRNA transcripts, which may be expressed by various tissue types, cellular phenotypes and developmental stages. Previous research suggests that there may be as many as 55,000 pre-miRNA encoding loci throughout the human genome^44^, which would mean, if equally distributed, that the MHC would be expected to harbor ~74 pre-miRNA hairpins. However, our computational estimates suggests the existence of many more pre-miRNA hairpins than anticipated within the MHC, suggesting that the gene dense MHC may harbor more miRNAs than previously calculated. It is only after we are able to characterize the totality of miRNA transcripts across a variety of tissue types, diseases and developmental states from diverse populations that we can begin to understand the full spectrum of miRNA diversity within the MHC. In order to properly study allele and haplotype specific miRNA expression patterns of MHC encoded miRNAs, it is first necessary to resolve the sequence of an individual’s MHC haplotype so that expressed transcripts can be accurately mapped to an individual’s MHC haplotype sequence. Working toward this goal, we have developed a long fragment DNA enrichment method^45^, capable of generating long DNA fragments that may be utilized by single molecule sequencing platforms to generate long reads, which has been previously utilized by our group for *de novo* assembly of the MHC^46^. These efforts lay the foundation for studying allele and haplotype specific transcript expression patterns across diverse sample populations and may be extended to any portion of the genome of interest.

Although determining the functional significance of each identified novel miRNA is beyond the scope of our current work, our data suggest that a subset of identified novel miRNA lie within LD of a disease associated SNP. It should also be noted that the vast majority of disease-associated variants found to be in LD with novel miRNA are located within non-coding regions of the MHC. Considering that 90% of causal autoimmune disease variants reside within non-coding regions of the genome^5^, it is possible that these miRNAs may play a role in the pathophysiology of the associated diseases and warrants further experimental investigation. Furthermore, five of the identified novel miRNAs share considerable sequence homology with oncogenic miRNAs (oncomiRs) including miR-590^29^, miR-196^27,28^, miR-489^26^, miR-508^30^ and miR-143^31^. These novel miRNAs are shown to have a high degree of sequence homology with already known and previously described annotated miRNAs (Fig. 3) and may be indicative of a shared target repertoire and redundant physiological function. Together, these data suggest that a subset of the identified novel miRNAs may contribute to the pathophysiology of numerous diseases, laying the groundwork for future studies to elucidate the functional connections of miRNAs to the numerous diseases associated with sequence variants within non-coding regions of the MHC.

## Methods and Materials

### Cell Culture

COX cells were obtained from the International Histocompatibility Working Group, Seattle, WA [(IHW09022) http://www.ihwg.org/hla/index.html]. PGF cells were obtained from the Coriell Biorepository (Cat #GM03107). They are both homozygous for chromosome 6. COX HLA typing is: HLA-A 01:01:01:01, HLA-B 08:01:01, HLA-C 07:01:01, HLA-DRB1 03:01:01:01, HLA-DQB1 02:01, HLA-DPB1 03:01. PGF HLA typing is: HLA-A 03:01:01:01, HLA-B 07:02:01, HLA-C 07:02:01:03, HLA-DRB1 15:01:01:01, HLA-DQB1 06:02:01, HLA-DPB1 04:01. Cells were cultured in RPMI-1640 medium with 15% FBS (Sigma Cat # F2442-500ML).

### Identifying Novel miRNA Transcripts of the MHC

Total RNA was extracted from two biological replicates (separate cell cultures collected at two individual time points) of PGF (n = 2) and COX (n = 2) cells using the Qiagen miRNeasy kit (Cat #217084) per manufacturer’s protocol. RNA was quantified on a Nanodrop ND-100 spectrophotometer, followed by RNA quality assessment on an Agilent 2200 TapeStation (Agilent Technologies, Palo Alto, CA). Library construction, workflow analysis and sequencing runs were performed following standard Illumina TruSeq Small RNA protocol (15004197 Revision G**)**. 50-base-pair single-end reads were generated on the Illumina NextSeq. 500 sequencing platform and stored in FASTQ format.

Raw sequencing reads from each cell line were mapped to a haplotype specific version of the reference genome (HG38) using Novoalign, allowing up to one mismatch per read as compared to the reference genome. Reads generated from PGF were mapped to the reference genome (GRCh38), which excluded all other MHC haplotype assemblies. Reads generated from COX were mapped to the reference genome (GRCh38), which included only the COX MHC haplotype assembly.

Novel miRNAs were identified from mapped reads using miRDeep* (version 36)^17^ generated from each biological replicate of PGF (n = 2) and COX (n = 2) cell lines independently. The following miRDeep* parameters were used: minimum phred score of 15, miRNA length of 16–28 (length range of miRNA within miRBase release 21^6^, max multi-map of 500, minimum score of −15 and minimum read depth of 1. Novel miRNAs were then further filtered, to include only those that are significantly expressed within each sequencing run, utilizing a previously implemented method^9^.

### Supporting Functional Evidence for Identified Novel miRNAs

41 Argonaut (Ago) CLIP-seq datasets from 4 independent studies^22–25^ were interrogated for the presence of the 89 identified novel mature miRNA sequences. The raw data (fastq) files from each dataset were interrogated in order to find the number of Ago supporting reads for each of the 89 novel miRNAs identified by our analysis. Only reads containing the exact, ungapped sequence of each mature miRNA were considered as supporting reads. The number of Ago CLIP-seq reads supporting each novel miRNA was tabulated. Only those miRNAs that were supported by 500 reads or more within all datasets were considered to be Ago Supported.

Dicer silencing was performed by designing a small hairpin RNA (shRNA) vector, which was subsequently transfected into a lentiviral plasmid for transduction into COX and PGF cells, effectively silencing Dicer expression by RNA interference (RNAi) within COX and PGF cells. The lentiviral plasmid containing the Dicer shRNA insert (GeneCopoeia catalog #HSH066175) was generated in cultured HEK293T cells by transfecting with a psi-LVRH1GP vector (GeneCopoeia). A lentiviral plasmid containing a “scrambled” sequence insert (i.e., randomized Dicer shRNA sequence) was similarly generated in HEK293T cells. Media was discarded after 24 hours post-transfection and packaging media was added to the plate. Scrambled and shRNA Dicer viruses were collected every 24 hours for 2 days. For transduction, 1.5 × 10^5^ COX and PGF cells were plated in 6 well plates, and 2 ml of fresh scrambled or Dicer silencing lentivirus was added along with 4 mg/ml polybrene. The plate was centrifuged at 2500 rpm for 90 minutes. After 10 hours, 2 ml of additional virus with polybrene was added and the plate was centrifuged at 2500 rpm for 90 minutes. After 16 hours, 2 ml of media was discarded and 2 ml of fresh virus and polybrene were added, and the plate was centrifuged at 2500 rpm for 90 minutes. Transduction was allowed to continue for an additional 24 hours before cells were collected for RNA extraction. RNA was extracted using the miRNeasy kit (Qiagen). Total RNA was reverse transcribed using the Qiagen miScript II RT kit (Cat #218160), with either (1) the miScript HiFlex Buffer (for quantification of Dicer mRNA) or (2) MiScript HiSpec Buffer (for specific quantification of mature miRNA only). Q-PCR was performed on cDNA generated by reverse transcription using a miSCRIPT SYBR Green PCR kit (Cat #21803). For the purposes of validating Dicer silencing, cDNA generated using the MiScript HiFlex buffer was quantified with Dicer specific qPCR primers (forward primer sequence: 5′-TTAACCTTTTGGTGTTTGATGAGTGT-3′, reverse primer sequence: 5′-GGACATGATGGACAATTTTCACA-3′). For the purpose of assessing the quantities of mature miRNA in both Dicer silencing and scrambled control conditions, cDNA generated using the MiScript HiSpec Buffer was quantified using miRNA specific primers. The primers were DNA oligos corresponding to the full length mature miRNA sequences. Sequences of mature miRNAs are included in supplemental Table 1. Primers were obtained from Qiagen and IDT. Two biological replicates for each condition were performed (silencing and control) and p-values were calculated using a t-test on normalized (beta-actin) ΔCt values^47^. Even though we tested various small nuclear RNAs as reference, they were not expressed in equivalent and reproducible amounts in COX and PGF cells, therefore beta-actin was chosen as providing reproducible and comparable expression in COX and PGF cells. A p-value less than 0.05 was deemed significant.

### Haplotype Conservation of Novel miRNAs

Novel miRNA sequence conservation across annotated MHC haplotype sequences was interrogated using BLAST. All eight available MHC haplotype sequences (PGF, COX, APD, SSTO, QBL, DBB, MANN and MCF) were scanned for the existence of every identified mature and pre-miRNA sequence (Supplemental Table 1) using BLAST (version 2.4.0+; parameters: -task megablast -word_size 7 -evalue 1000)^48^. Only perfectly matched sequences (100% sequence identity between the query sequence and the reference MHC haplotype) were considered to be conserved.

### Sequence Homology of Novel miRNAs to Known miRNAs

The target specificity of a miRNA transcript is primarily determined by its sequence, which directs the formation of an energetically favorable double stranded RNA heteroduplex between the miRNA and a complementary RNA target sequence^49,50^. Consequently, sequence homology amongst miRNA transcripts may be indicative of a shared target repertoire and redundant physiological function. In order to evaluate the sequence homology between each of the identified novel miRNA transcripts (n = 89) and all annotated miRNAs within miRBase (release 21), each novel miRNA sequence was aligned pairwise with every annotated miRNA sequence using the semi-global Needleman-Wunsch algorithm implemented within MATLAB (2014a). For each identified novel miRNA, the closest matched annotated miRNA (highest alignment score) was reported. In the case in which a novel miRNA aligned to multiple annotated miRNA with the same maximal alignment score, every match was reported (Supplemental Table 2).

### *In Silico* Discovery of Putative Pre-miRNA Encoding Loci

A computational pipeline was developed (Fig. 4) in order to identify every putative pre-miRNA encoding locus present within the reference MHC haplotype sequences of both PGF and COX^2^. The developed pipeline takes a FASTA file as input, which was generated from the reference genome (HG19) using BEDtools^51,52^ and ranged from HG38 coordinates chr6:28510019-33383765 and chr6_cox_hap2:1-4795371 for PGF and COX haplotypes respectively. The pipeline begins by first partitioning the FASTA file provided as input into overlapping segments of variable length using a 1 bp sliding window (58 ≤ Length ≥ 110), generating sequences from both the forward and reverse strands (output in the 5′− > 3′ direction) for each iteration so as to enumerate every possible pre-miRNA encoding locus throughout the MHC. *In silico* RNA folding was subsequently performed for each putative pre-miRNA transcript to determine the minimum free energy (MFE) and secondary structure of each theoretical transcript using RNAfold^53^. The acceptable parameter range for both window length (pre-miRNA transcript length) and MFE were selected as the 95^th^ and 5^th^ percentile value for both distributions of annotated human pre-miRNA within miRbase release 21 (n = 1,881)^6^, corresponding to a length of 58–110 bp and a maximum MFE of −20Kcal/mol respectively. In order to further reduce the search space, pre-miRNA hairpin structures containing bulge loops or multi-stem structures and those with a MFE ≥ −20 Kcal/mol were removed, leaving only characteristic linear pre-miRNA hairpin with a permissible MFE. The machine-learning algorithm, miRBoost^54^ was then utilized to identify high confidence pre-miRNA hairpin structures. The miRBoost positive and negative control training sets comprised of the human pre-miRNA present within miRBase (release 21) and the provided internal miRBoost negative Human control dataset respectively. Lastly, the BED formatted output file was subsequently filtered to remove any entries that overlap an annotated exon (GENCODE v24)^55^ and the remaining entries then merged so as to remove overlapping entries and create an atlas of loci throughout the MHC that contain at least one putative pre-miRNA hairpin locus.

BLAST was used to determine sequence homology amongst computationally predicted pre-miRNA encoding loci identified from the *in silico* analysis of both the PGF (n = 9019) and COX (n = 9207) MHC haplotypes. The sequences of each putative pre-miRNA encoding loci identified from both haplotypes were aligned pairwise using BLAST and filtered to include only those loci whose sequences matched 100% (minimum overlap defined by the shortest of the two compared sequences).

### Novel miRNA Loci within LD of Disease Associated SNPs

Annotated disease associated SNPs within the MHC were collected from the GWAS catalog (www.ebi.ac.uk/gwas, accessed on March 1, 2017)^32,33^. The linkage disequilibrium (LD) block of each disease associated SNP was calculated using SNAP (HapMap release 22) with a minimum r^2^ of 0.9^56^. The LD blocks defined by each disease associated SNP were then intersected with the set of empirically derived novel miRNA as well as the set of identified computationally predicted pre-miRNA encoding loci using BEDtools^51,52^ in order to determine which novel miRNA encoding loci lie within the LD block of each disease associated SNP.

## Electronic supplementary material

Supplemental Material
