# Supplementary material for: Novel and Haplotype Specific MicroRNAs Encoded by the Major Histocompatibility Complex
Source: Sci Rep. 2018 Mar 1;8:3832. doi: 10.1038/s41598-018-19427-6 (PMC5832780; doi:10.1038/s41598-018-19427-6)
Supplement: Supplementary file 1 — Supplemental Material [file 41598_2018_19427_MOESM1_ESM.doc]

**Supplemental Material**

**Novel and Haplotype Specific MicroRNAs Encoded by the Major**

**Histocompatibility Complex**

Clark PM1, Chitnis N1, Kamoun M2, Johnson B2, Monos D1,2,*

1Department of Pathology and Laboratory Medicine, The Children's Hospital of Philadelphia, Philadelphia, PA, 19104, USA.

2Department of Pathology and Laboratory Medicine, Perelman School of Medicine,

University of Pennsylvania, Philadelphia, PA, 19104, USA.

*Correspondence to: Dimitri Monos, monosd@email.chop.edu.

| **Novel miRNA ID** | **miRDeep* Score** | **chr** | **strand** | **hairPin_loci** | **mature_loci** | **Mature miRNA Sequence** | **Dicer Dependent** | **AGO Supported** | **Cell Line Expression** |
| --- | --- | --- | --- | --- | --- | --- | --- | --- | --- |
| CHOP_1 | -1.64 | chr6 | - | 28869349-28869431 | 28869398-28869421 | aacccaggaggcggaacuugcagu | Yes | Yes | PGF & COX |
| CHOP_2 | 0.26 | chr6 | - | 31533661-31533735 | 31533671-31533690 | aauagccacugcacuccagc | Yes | Yes | PGF & COX |
| CHOP_3 | -3.39 | chr6 | - | 30593611-30593683 | 30593621-30593639 | acccaggaggcagagguug |  | Yes | PGF & COX |
| CHOP_4 | -1.2 | chr6 | + | 31686084-31686168 | 31686134-31686158 | acugcacuccagccugggcaacaua | Yes | Yes | PGF & COX |
| CHOP_5 | -3.49 | chr6 | - | 33373716-33373786 | 33373759-33373776 | aggcuggagugcaauggc | Yes | Yes | PGF & COX |
| CHOP_6 | 0.08 | chr6 | + | 31922603-31922681 | 31922613-31922634 | agguggaucaccugaggucagg |  | Yes | PGF & COX |
| CHOP_7 | -9.36 | chr6 | - | 32078526-32078594 | 32078536-32078552 | aguacuuggaugggaga |  | Yes | PGF & COX |
| CHOP_8 | -7.61 | chr6 | + | 30122198-30122276 | 30122245-30122266 | aguucgagaccagccugggcaa |  | Yes | PGF & COX |
| CHOP_9 | 109.99 | chr6 | + | 30773502-30773578 | 30773548-30773568 | cacugcacuccagccugggca |  | Yes | PGF & COX |
| CHOP_10 | -1.4 | chr6 | + | 33226125-33226203 | 33226172-33226193 | cacugcacucuagccugggcga |  | Yes | PGF & COX |
| CHOP_11 | -10.07 | chr6 | - | 32118342-32118412 | 32118385-32118402 | caggcuggucucgaacuc |  | Yes | PGF & COX |
| CHOP_12 | -2.83 | chr6 | + | 31787739-31787813 | 31787784-31787803 | cugggcaacauagcgagacc |  | Yes | PGF & COX |
| CHOP_13 | 1.34 | chr6 | - | 30111222-30111296 | 30111232-30111251 | cugggcaacauagcgagacu | Yes | Yes | PGF & COX |
| CHOP_14 | 103.45 | chr6 | + | 31757155-31757225 | 31757198-31757215 | gcaguggcgcgaucucgg |  | Yes | PGF & COX |
| CHOP_15 | 349.19 | chr6 | + | 32981887-32981957 | 32981930-32981947 | gccgagaucgcgccacug | Yes | Yes | PGF & COX |
| CHOP_16 | -13.02 | chr6 | - | 30530520-30530600 | 30530568-30530590 | ggaggaucgcuugaguccaggag |  | Yes | PGF & COX |
| CHOP_17 | -1.37 | chr6 | - | 28673839-28673917 | 28673886-28673907 | ggggauguagcucagugguaga |  | Yes | PGF & COX |
| CHOP_18 | -2.12 | chr6 | + | 28719694-28719776 | 28719704-28719727 | ggggguguagcucagugguagagc | Yes | Yes | PGF & COX |
| CHOP_19 | 0.76 | chr6 | + | 30955647-30955713 | 30955688-30955703 | gucccggcggagucgc | Yes | Yes | PGF & COX |
| CHOP_20 | -4.19 | chr6 | - | 29597099-29597169 | 29597142-29597159 | guugcccaggcuggagug | Yes | Yes | PGF & COX |
| CHOP_21 | 229.13 | chr6 | + | 33201670-33201750 | 33201718-33201740 | uacuugaccuugacucucccuca | Yes |  | PGF & COX |
| CHOP_22 | -2.86 | chr6 | + | 33368155-33368233 | 33368202-33368223 | ugaccucgugaucugcccgccu | Yes | Yes | PGF & COX |
| CHOP_23 | -7.23 | chr6 | + | 31666746-31666830 | 31666796-31666820 | uuuacugacacuguucuuuuucuag | Yes |  | PGF & COX |
| CHOP_24 | -11.91 | chr6 | - | 28822284-28822364 | 28822332-28822354 | uuuaguagagacgggguuucacc |  | Yes | PGF & COX |
| CHOP_25 | -6.68 | chr6 | - | 30984488-30984566 | 30984498-30984519 | uuuuguauuuuuaguagagaca | Yes | Yes | PGF & COX |
| CHOP_26 | 1.88 | chr6 | - | 32621704-32621782 | 32621714-32621735 | aauuucugcauaguccaccucu | Yes |  | PGF |
| CHOP_27 | -2.15 | chr6 | + | 32060665-32060745 | 32060713-32060735 | acugcaaccucugccucccgggu |  | Yes | PGF |
| CHOP_28 | -4.58 | chr6 | + | 31783638-31783708 | 31783681-31783698 | agacggagucucgcucug |  | Yes | PGF |
| CHOP_29 | -4.79 | chr6 | - | 29616609-29616689 | 29616657-29616679 | agagucuugcucuguugccuagg |  | Yes | PGF |
| CHOP_30 | -4.19 | chr6 | - | 33381575-33381661 | 33381585-33381610 | aggcaggagaaucacuugaacccggg | Yes | Yes | PGF |
| CHOP_31 | -2.83 | chr6 | - | 31705212-31705290 | 31705259-31705280 | aggcggaucaccugaggucagg |  | Yes | PGF |
| CHOP_32 | -2.2 | chr6 | + | 31595059-31595149 | 31595112-31595139 | aggcugaggcaggagaaucacuugaacc | Yes | Yes | PGF |
| CHOP_33 | -5.82 | chr6 | + | 31555271-31555341 | 31555281-31555298 | aggcuggagugcacuggc |  | Yes | PGF |
| CHOP_34 | -1.61 | chr6 | + | 29983639-29983717 | 29983686-29983707 | aguucgagaccagccugaccaa | Yes | Yes | PGF |
| CHOP_35 | -2.86 | chr6 | - | 30726532-30726608 | 30726578-30726598 | caacauagcaagacuccaucu | Yes | Yes | PGF |
| CHOP_36 | -2.2 | chr6 | + | 33219323-33219397 | 33219333-33219352 | cacccaggcuggagagcagu |  | Yes | PGF |
| CHOP_37 | 0.42 | chr6 | + | 33258608-33258692 | 33258658-33258682 | cacugcaaccuccgccucccagguu |  | Yes | PGF |
| CHOP_38 | 1.02 | chr6 | + | 30243415-30243491 | 30243461-30243481 | cacugcacuccagccugggcg | Yes | Yes | PGF |
| CHOP_39 | 0.62 | chr6 | + | 31527950-31528030 | 31527998-31528020 | cagccugggcaacagagcgagac | Yes | Yes | PGF |
| CHOP_40 | -1.68 | chr6 | - | 32893643-32893717 | 32893688-32893707 | cagggucucgcucugucgcc |  | Yes | PGF |
| CHOP_41 | 0.72 | chr6 | + | 29188931-29189015 | 29188981-29189005 | caugugucuuuauaguagaaugauu | Yes | Yes | PGF |
| CHOP_42 | 12.11 | chr6 | + | 30773501-30773577 | 30773547-30773567 | ccacugcacuccagccugggc | Yes | Yes | PGF |
| CHOP_43 | 73.62 | chr6 | - | 32441274-32441344 | 32441317-32441334 | ccaggcuggagugcagug | Yes | Yes | PGF |
| CHOP_44 | -3.35 | chr6 | - | 32885544-32885618 | 32885554-32885573 | cccagcuacucgggaggcug | Yes | Yes | PGF |
| CHOP_45 | -1.65 | chr6 | - | 32878435-32878515 | 32878483-32878505 | cccgggaggcggagcuugcagug | Yes | Yes | PGF |
| CHOP_46 | -1.27 | chr6 | - | 32203130-32203210 | 32203178-32203200 | cgggcacaguggcucacgccugu |  | Yes | PGF |
| CHOP_47 | -1.63 | chr6 | - | 30902270-30902350 | 30902318-30902340 | cucacgccuguaaucccagcacc | Yes | Yes | PGF |
| CHOP_48 | -6.95 | chr6 | - | 31703910-31703982 | 31703920-31703938 | cucacugcaaccucugccu | Yes | Yes | PGF |
| CHOP_49 | -3.18 | chr6 | + | 28764408-28764488 | 28764418-28764440 | cugaagaucuaaaggucccuggu |  |  | PGF |
| CHOP_50 | -5.11 | chr6 | - | 28807841-28807923 | 28807851-28807874 | cugaagaucuaaaggucccugguu | Yes |  | PGF |
| CHOP_51 | -3.14 | chr6 | - | 30117417-30117487 | 30117427-30117444 | cugaccucgugauccgcc |  | Yes | PGF |
| CHOP_52 | 27.11 | chr6 | + | 32326294-32326374 | 32326342-32326364 | gaggacuguauuugugacuaauu | Yes | Yes | PGF |
| CHOP_53 | -13.18 | chr6 | - | 29783083-29783167 | 29783093-29783117 | gagguguuuauaguauucucugaug |  | Yes | PGF |
| CHOP_54 | -4.58 | chr6 | - | 33160492-33160564 | 33160502-33160520 | gagucucgcucugucgccc | Yes | Yes | PGF |
| CHOP_55 | 56.6 | chr6 | + | 28709145-28709227 | 28709194-28709217 | gccaagaucgcgccacugcacucc | Yes | Yes | PGF |
| CHOP_56 | 0.33 | chr6 | + | 31901998-31902070 | 31902042-31902060 | gcgcgcggcggcggcggcg |  | Yes | PGF |
| CHOP_57 | -1.04 | chr6 | - | 30648625-30648695 | 30648668-30648685 | gcucacgccuguaauccc | Yes | Yes | PGF |
| CHOP_58 | -7.08 | chr6 | + | 28934513-28934599 | 28934564-28934589 | gcugaggcaggagaaucgcuugaacc | Yes | Yes | PGF |
| CHOP_59 | -0.8 | chr6 | - | 28827416-28827494 | 28827463-28827484 | ggggguauagcucagugguaga | Yes | Yes | PGF |
| CHOP_60 | -8.1 | chr6 | - | 32078492-32078566 | 32078537-32078556 | gguuaguacuuggaugggag | Yes | Yes | PGF |
| CHOP_61 | -3.86 | chr6 | + | 31651475-31651545 | 31651485-31651502 | gucgagaucgcgccacug | Yes | Yes | PGF |
| CHOP_62 | -4.83 | chr6 | - | 28777850-28777930 | 28777860-28777882 | uaauuuuuuguauuuuuaguaga |  | Yes | PGF |
| CHOP_63 | -2.28 | chr6 | - | 33232017-33232087 | 33232027-33232044 | ucacugcaaccuccgccu |  | Yes | PGF |
| CHOP_64 | 30.55 | chr6 | + | 32124871-32124953 | 32124920-32124943 | ucacugcaagcuccgccucccggg | Yes | Yes | PGF |
| CHOP_65 | -3.8 | chr6 | - | 31719620-31719704 | 31719630-31719654 | ugaggcaggagaaucgcuugaaccu | Yes | Yes | PGF |
| CHOP_66 | -7.52 | chr6 | - | 32517790-32517868 | 32517800-32517821 | uugaaagagaggaaaagaagcu | Yes |  | PGF |
| CHOP_67 | 0.26 | chr6 | - | 28752429-28752507 | 28752476-28752497 | uuggccaggcuggucucgaacu | Yes | Yes | PGF |
| CHOP_68 | -12.05 | chr6_cox_hap2 | + | 1203973-1204049 | 1204019-1204039 | aauuuuuguauuuuugguaga |  | Yes | COX |
| CHOP_69 | 0.33 | chr6_cox_hap2 | - | 4674763-4674837 | 4674808-4674827 | acccaggcuggagugcagug |  | Yes | COX |
| CHOP_70 | -1.42 | chr6_cox_hap2 | - | 4739873-4739953 | 4739883-4739905 | aggcaggagaauugcuugaaccc | Yes | Yes | COX |
| CHOP_71 | -0.04 | chr6_cox_hap2 | - | 2173243-2173321 | 2173253-2173274 | agugcaguggcgcgaucucggc |  | Yes | COX |
| CHOP_72 | -0.9 | chr6_cox_hap2 | - | 3394317-3394387 | 3394360-3394377 | cccaggcuggaguacagu | Yes | Yes | COX |
| CHOP_73 | -1.72 | chr6_cox_hap2 | - | 2206377-2206453 | 2206423-2206443 | cgacauagcaagacuccaucu |  |  | COX |
| CHOP_74 | -2.23 | chr6_cox_hap2 | + | 219418-219494 | 219428-219448 | cgcccaggcuggagugcagug |  | Yes | COX |
| CHOP_75 | 1.87 | chr6_cox_hap2 | - | 3217879-3217955 | 3217889-3217909 | cgccccaggucucggucccug | Yes |  | COX |
| CHOP_76 | -1.48 | chr6_cox_hap2 | - | 2662534-2662620 | 2662544-2662569 | cugaggugggaggaucgcuugagccu | Yes | Yes | COX |
| CHOP_77 | -7.3 | chr6_cox_hap2 | - | 270979-271049 | 271022-271039 | cugggacuacaggcaccc | Yes | Yes | COX |
| CHOP_78 | -3.37 | chr6_cox_hap2 | - | 3229400-3229470 | 3229443-3229460 | gagucuugcucugucgcc | Yes | Yes | COX |
| CHOP_79 | 0.33 | chr6_cox_hap2 | + | 3379533-3379601 | 3379575-3379591 | gcgcggcggcggcggcg | Yes | Yes | COX |
| CHOP_80 | -3.74 | chr6_cox_hap2 | - | 2413252-2413318 | 2413262-2413277 | gcuacucgggaggcug | Yes | Yes | COX |
| CHOP_81 | -8.22 | chr6_cox_hap2 | + | 3497783-3497853 | 3497793-3497810 | gcugagaucacaccacug | Yes | Yes | COX |
| CHOP_82 | -5.49 | chr6_cox_hap2 | + | 4650556-4650630 | 4650601-4650620 | ggccgggcaugguggcucac | Yes | Yes | COX |
| CHOP_83 | 177.22 | chr6_cox_hap2 | - | 226217-226295 | 226264-226285 | ggguguagcucagugguagagc | Yes | Yes | COX |
| CHOP_84 | -5.37 | chr6_cox_hap2 | + | 3413207-3413277 | 3413217-3413234 | gucucgaacuccugaccu | Yes | Yes | COX |
| CHOP_85 | -1.27 | chr6_cox_hap2 | - | 1597329-1597407 | 1597339-1597360 | uccugaccucgugauccgcccg |  | Yes | COX |
| CHOP_86 | -2.65 | chr6_cox_hap2 | - | 3089988-3090066 | 3089998-3090019 | ugcugggauuacaggugugagc | Yes | Yes | COX |
| CHOP_87 | -2.52 | chr6_cox_hap2 | - | 4297806-4297894 | 4297858-4297884 | uguagucccagcuacucgggaggcuga | Yes | Yes | COX |
| CHOP_88 | -10.98 | chr6_cox_hap2 | + | 4456674-4456756 | 4456684-4456707 | uugauuugcauuucucugauggcc |  | Yes | COX |
| CHOP_89 | -12.15 | chr6_cox_hap2 | + | 55320-55390 | 55363-55380 | uuuguauuuuuaguagag |  | Yes | COX |

**Supplemental Table 1.** Identified novel miRNA of the MHC derived from the analysis of RNA-Seq data. For each miRNA, the identified locus of both the precursor and mature miRNA is provided in addition to the mature miRNA sequence. For those miRNAs found to be expressed within both PGF and COX haplotypes the PGF coordinates are listed. Functional evidence including whether or not the biogenesis of the mature miRNA was found to be Dicer dependent and whether or not the mature miRNA was found to be loaded onto the Ago silencing complex is also provided along with the cell line which was found to express the novel miRNA.

| **Novel miRNA ID** | **miRBase ID** | **Alignment Score** | **Percent Identity** |
| --- | --- | --- | --- |
| CHOP_1 | hsa-miR-1254 | 46.3 | 75.0 |
| CHOP_2 | hsa-miR-1273g-3p | 48.3 | 75.0 |
| CHOP_3 | hsa-miR-7851-3p | 39.7 | 78.9 |
| CHOP_4 | hsa-miR-1273g-3p | 51.0 | 68.0 |
| CHOP_5 | hsa-miR-6781-5p | 34.3 | 72.2 |
| CHOP_6 | hsa-miR-6853-5p | 36.0 | 81.8 |
| CHOP_7 | hsa-miR-3156-5p | 27.0 | 76.5 |
| CHOP_8 | hsa-miR-584-3p | 41.3 | 68.2 |
| CHOP_9 | hsa-miR-1273g-3p | 55.3 | 85.7 |
| CHOP_10 | hsa-miR-1273g-3p | 50.7 | 77.3 |
| CHOP_11 | hsa-miR-1469 | 36.3 | 66.7 |
| CHOP_12 | hsa-miR-1285-3p | 49.0 | 90.0 |
| CHOP_13 | hsa-miR-1285-3p | 44.3 | 85.0 |
| CHOP_14 | hsa-miR-323a-5p | 37.3 | 72.2 |
| CHOP_15 | hsa-miR-6850-3p | 38.0 | 77.8 |
| CHOP_16 | hsa-miR-3978 | 37.7 | 65.2 |
| CHOP_17 | hsa-miR-128-1-5p | 35.3 | 68.2 |
| CHOP_18 | hsa-miR-5001-5p | 40.3 | 70.8 |
| CHOP_19 | hsa-miR-4745-3p | 39.3 | 75.0 |
| CHOP_20 | hsa-miR-5006-5p | 35.3 | 77.8 |
| CHOP_21 | hsa-miR-6759-3p | 47.7 | 78.3 |
| CHOP_22 | hsa-miR-3150b-5p | 44.3 | 72.7 |
| CHOP_23 | hsa-miR-6878-3p | 37.0 | 68.0 |
| CHOP_24 | hsa-miR-1909-3p | 35.3 | 60.9 |
| CHOP_24 | hsa-miR-298 | 35.3 | 65.2 |
| CHOP_25 | hsa-miR-544a | 26.3 | 63.6 |
| CHOP_26 | hsa-miR-6849-3p | 38.7 | 59.1 |
| CHOP_27 | hsa-miR-1273h-3p | 52.3 | 69.6 |
| CHOP_28 | hsa-miR-1303 | 40.3 | 83.3 |
| CHOP_29 | hsa-miR-1285-5p | 40.3 | 73.9 |
| CHOP_30 | hsa-miR-7974 | 43.7 | 61.5 |
| CHOP_31 | hsa-miR-4746-3p | 40.0 | 59.1 |
| CHOP_32 | hsa-miR-7974 | 39.3 | 53.6 |
| CHOP_33 | hsa-miR-5589-5p | 35.7 | 83.3 |
| CHOP_34 | hsa-miR-584-3p | 41.0 | 63.6 |
| CHOP_35 | hsa-miR-1273a | 46.0 | 81.0 |
| CHOP_36 | hsa-miR-941 | 40.3 | 80.0 |
| CHOP_37 | hsa-miR-6805-3p | 57.7 | 68.0 |
| CHOP_38 | hsa-miR-1273g-3p | 55.3 | 85.7 |
| CHOP_39 | hsa-miR-1285-3p | 44.3 | 73.9 |
| CHOP_40 | hsa-miR-4512 | 48.0 | 80.0 |
| CHOP_41 | hsa-miR-8057 | 29.0 | 56.0 |
| CHOP_42 | hsa-miR-1273g-3p | 59.7 | 90.5 |
| CHOP_43 | hsa-miR-3135a | 39.7 | 94.4 |
| CHOP_44 | hsa-miR-3194-5p | 42.0 | 80.0 |
| CHOP_45 | hsa-miR-1254 | 46.7 | 78.3 |
| CHOP_46 | hsa-miR-4665-3p | 46.3 | 65.2 |
| CHOP_47 | hsa-miR-3620-3p | 50.3 | 78.3 |
| CHOP_48 | hsa-miR-6511b-3p | 44.3 | 73.7 |
| CHOP_49 | hsa-miR-127-5p | 40.0 | 73.9 |
| CHOP_50 | hsa-miR-127-5p | 40.0 | 70.8 |
| CHOP_51 | hsa-miR-1908-3p | 41.3 | 66.7 |
| CHOP_52 | hsa-miR-489-5p | 30.3 | 69.6 |
| CHOP_53 | hsa-miR-653-5p | 29.3 | 60.0 |
| CHOP_54 | hsa-miR-3184-3p | 44.7 | 84.2 |
| CHOP_55 | hsa-miR-4695-3p | 47.7 | 62.5 |
| CHOP_56 | hsa-miR-3960 | 45.3 | 84.2 |
| CHOP_57 | hsa-miR-1470 | 40.7 | 66.7 |
| CHOP_58 | hsa-miR-7974 | 38.7 | 57.7 |
| CHOP_59 | hsa-miR-1225-5p | 37.3 | 68.2 |
| CHOP_60 | hsa-miR-5087 | 29.0 | 80.0 |
| CHOP_61 | hsa-miR-196b-3p | 36.0 | 72.2 |
| CHOP_62 | hsa-miR-590-3p | 25.7 | 69.6 |
| CHOP_63 | hsa-miR-6727-3p | 44.0 | 77.8 |
| CHOP_64 | hsa-miR-1273h-3p | 52.7 | 66.7 |
| CHOP_65 | hsa-miR-7974 | 38.3 | 60.0 |
| CHOP_66 | hsa-miR-6795-5p | 30.3 | 63.6 |
| CHOP_66 | hsa-miR-8085 | 30.3 | 63.6 |
| CHOP_67 | hsa-miR-6508-3p | 41.0 | 72.7 |
| CHOP_68 | hsa-miR-508-3p | 24.3 | 71.4 |
| CHOP_69 | hsa-miR-3135a | 42.0 | 85.0 |
| CHOP_70 | hsa-miR-7974 | 38.3 | 65.2 |
| CHOP_71 | hsa-miR-143-5p | 39.3 | 81.8 |
| CHOP_72 | hsa-miR-1291 | 36.7 | 66.7 |
| CHOP_72 | hsa-miR-1266-5p | 36.7 | 83.3 |
| CHOP_72 | hsa-miR-3135a | 36.7 | 83.3 |
| CHOP_73 | hsa-miR-1273a | 48.7 | 85.7 |
| CHOP_74 | hsa-miR-3135a | 44.3 | 85.7 |
| CHOP_75 | hsa-miR-6862-3p | 48.7 | 76.2 |
| CHOP_76 | hsa-miR-7974 | 40.3 | 65.4 |
| CHOP_77 | hsa-miR-4515 | 41.7 | 83.3 |
| CHOP_78 | hsa-miR-636 | 36.7 | 77.8 |
| CHOP_79 | hsa-miR-3960 | 43.7 | 88.2 |
| CHOP_80 | hsa-miR-3130-3p | 34.7 | 75.0 |
| CHOP_81 | hsa-miR-6857-3p | 37.7 | 72.2 |
| CHOP_82 | hsa-miR-1972 | 46.7 | 80.0 |
| CHOP_83 | hsa-miR-5001-5p | 38.7 | 77.3 |
| CHOP_84 | hsa-miR-3184-3p | 37.0 | 72.2 |
| CHOP_84 | hsa-miR-6793-3p | 37.0 | 66.7 |
| CHOP_85 | hsa-miR-1908-3p | 47.7 | 68.2 |
| CHOP_86 | hsa-miR-619-5p | 47.3 | 86.4 |
| CHOP_87 | hsa-miR-5585-5p | 50.7 | 70.4 |
| CHOP_88 | hsa-miR-7107-3p | 37.3 | 79.2 |
| CHOP_89 | hsa-miR-544a | 22.7 | 77.8 |

**Supplemental Table 2.** Each novel miRNA is reported along with the closest matched annotated miRNA sequence from miRBase (release 21) along with the alignment score and percentage identity between the two sequences.

| **Associated Disease / Trait** | **Disease Associated SNP** | **Genomic Context** | **Novel miRNA ID** |
| --- | --- | --- | --- |
| Age-related hearing impairment | rs6904029 | non coding transcript exon variant | CHOP_34 |
| Age-related macular degeneration | rs12153855 | intron variant | CHOP_11 |
|  |  |  | CHOP_64 |
|  | rs2071277 | intron variant | CHOP_46 |
| Antinuclear antibody levels | rs2395185 | intron variant | CHOP_66 |
| Arthritis (juvenile idiopathic) | rs2395148 | intron variant | CHOP_52 |
| Asthma | rs3129943 | intron variant | CHOP_52 |
| Atopic dermatitis | rs12153855 | intron variant | CHOP_11 |
|  |  |  | CHOP_64 |
| Autism spectrum disorder | rs3132581 | intron variant | CHOP_19 |
|  |  |  | CHOP_25 |
|  |  |  | CHOP_42 |
|  |  |  | CHOP_47 |
|  |  |  | CHOP_9 |
| Bipolar disorder and schizophrenia | rs2524005 | upstream gene variant | CHOP_34 |
|  | rs886424 | non coding transcript exon variant | CHOP_42 |
|  |  |  | CHOP_47 |
|  |  |  | CHOP_9 |
| Blood metabolite ratios | rs1046080 | missense variant | CHOP_23 |
|  |  |  | CHOP_4 |
|  |  |  | CHOP_61 |
| Cholesterol, total | rs3177928 | 3' UTR variant | CHOP_43 |
| Complement C3 and C4 levels | rs11575839 | synonymous variant | CHOP_2 |
|  |  |  | CHOP_33 |
|  |  |  | CHOP_39 |
|  | rs2071278 | intron variant | CHOP_11 |
|  |  |  | CHOP_27 |
|  |  |  | CHOP_46 |
|  |  |  | CHOP_60 |
|  |  |  | CHOP_64 |
|  |  |  | CHOP_7 |
| Crohns disease | rs1799964 | upstream gene variant | CHOP_32 |
|  | rs9258260 | upstream gene variant | CHOP_53 |
|  | rs9271366 | intergenic variant | CHOP_26 |
|  |  |  | CHOP_43 |
|  |  |  | CHOP_52 |
|  |  |  | CHOP_66 |
| Cutaneous lupus erythematosus | rs3094067 | intron variant | CHOP_13 |
|  |  |  | CHOP_16 |
|  |  |  | CHOP_34 |
|  |  |  | CHOP_38 |
|  |  |  | CHOP_51 |
|  |  |  | CHOP_8 |
|  | rs3094084 | upstream gene variant | CHOP_25 |
|  | rs3131060 | downstream gene variant | CHOP_42 |
|  |  |  | CHOP_47 |
|  |  |  | CHOP_9 |
|  | rs9267531 | non coding transcript exon variant | CHOP_12 |
|  |  |  | CHOP_14 |
|  |  |  | CHOP_23 |
|  |  |  | CHOP_28 |
|  |  |  | CHOP_31 |
|  |  |  | CHOP_4 |
|  |  |  | CHOP_48 |
|  |  |  | CHOP_56 |
|  |  |  | CHOP_61 |
|  |  |  | CHOP_65 |
| Diastolic blood pressure | rs805303 | intron variant | CHOP_23 |
|  |  |  | CHOP_61 |

| Disc degeneration (lumbar) | rs10046257 | regulatory region variant | CHOP_40 |
| --- | --- | --- | --- |
|  | rs10214886 | intergenic variant | CHOP_40 |
|  | rs1029295 | intergenic variant | CHOP_40 |
|  | rs1029296 | intergenic variant | CHOP_40 |
|  | rs11969002 | upstream gene variant | CHOP_40 |
|  | rs3749982 | upstream gene variant | CHOP_40 |
|  | rs6457690 | intergenic variant | CHOP_40 |
|  | rs6936004 | intergenic variant | CHOP_40 |
|  | rs7744666 | upstream gene variant | CHOP_40 |
|  | rs9469300 | upstream gene variant | CHOP_40 |
| Drug-induced liver injury (amoxicillin-clavulanate) | rs2523822 | intergenic variant | CHOP_34 |
| Emphysema imaging phenotypes | rs2070600 | missense variant | CHOP_11 |
|  |  |  | CHOP_12 |
|  |  |  | CHOP_14 |
|  |  |  | CHOP_27 |
|  |  |  | CHOP_28 |
|  |  |  | CHOP_46 |
|  |  |  | CHOP_56 |
|  |  |  | CHOP_6 |
|  |  |  | CHOP_60 |
|  |  |  | CHOP_64 |
|  |  |  | CHOP_65 |
|  |  |  | CHOP_7 |
| Epstein Barr virus nuclear antigen 1 IgG levels | rs2516049 | intron variant | CHOP_66 |
| Febrile seizures (MMR vaccine-related) | rs3130618 | missense variant | CHOP_61 |
| Graves disease | rs4313034 | non coding transcript exon variant | CHOP_34 |
| Hematology traits | rs389884 | non coding transcript exon variant | CHOP_11 |
|  |  |  | CHOP_12 |
|  |  |  | CHOP_14 |
|  |  |  | CHOP_27 |
|  |  |  | CHOP_28 |
|  |  |  | CHOP_46 |
|  |  |  | CHOP_56 |
|  |  |  | CHOP_6 |
|  |  |  | CHOP_60 |
|  |  |  | CHOP_64 |
|  |  |  | CHOP_7 |
| Hepatitis B vaccine response | rs9267665 | intron variant | CHOP_12 |
|  |  |  | CHOP_14 |
|  |  |  | CHOP_23 |
|  |  |  | CHOP_28 |
|  |  |  | CHOP_31 |
|  |  |  | CHOP_4 |
|  |  |  | CHOP_48 |
|  |  |  | CHOP_56 |
|  |  |  | CHOP_61 |
|  |  |  | CHOP_65 |
| Hepatitis C induced liver cirrhosis | rs3129860 | intron variant | CHOP_26 |
|  | rs3129860 | intron variant | CHOP_43 |
|  | rs3129860 | intron variant | CHOP_52 |
|  | rs3129860 | intron variant | CHOP_66 |
| HIV-1 control | rs12198173 | intron variant | CHOP_27 |
|  | rs12198173 | intron variant | CHOP_60 |
|  | rs12198173 | intron variant | CHOP_7 |
|  | rs7756521 | intron variant | CHOP_47 |
|  | rs9368699 | 5' UTR variant | CHOP_56 |
|  | rs9368699 | 5' UTR variant | CHOP_6 |
| Hodgkins lymphoma | rs2395185 | intron variant | CHOP_66 |

| Hypertension | rs805303 | intron variant | CHOP_23 |
| --- | --- | --- | --- |
|  |  |  | CHOP_61 |
| Idiopathic membranous nephropathy | rs3115663 | non coding transcript exon variant | CHOP_61 |
|  | rs3129939 | intron variant | CHOP_52 |
|  | rs3130618 | missense variant | CHOP_61 |
|  | rs3132580 | missense variant | CHOP_19 |
|  |  |  | CHOP_25 |
|  |  |  | CHOP_42 |
|  |  |  | CHOP_47 |
|  |  |  | CHOP_9 |
|  | rs3134792 | intergenic variant | CHOP_2 |
|  |  |  | CHOP_39 |
|  | rs389884 | non coding transcript exon variant | CHOP_11 |
|  |  |  | CHOP_12 |
|  |  |  | CHOP_14 |
|  |  |  | CHOP_27 |
|  |  |  | CHOP_28 |
|  |  |  | CHOP_46 |
|  |  |  | CHOP_56 |
|  |  |  | CHOP_6 |
|  |  |  | CHOP_60 |
|  |  |  | CHOP_64 |
|  |  |  | CHOP_7 |
|  | rs7775397 | missense variant | CHOP_11 |
|  |  |  | CHOP_26 |
|  |  |  | CHOP_27 |
|  |  |  | CHOP_43 |
|  |  |  | CHOP_46 |
|  |  |  | CHOP_52 |
|  |  |  | CHOP_60 |
|  |  |  | CHOP_64 |
|  |  |  | CHOP_66 |
|  |  |  | CHOP_7 |
| IgG glycosylation | rs16871226 | intron variant | CHOP_15 |
|  |  |  | CHOP_40 |
|  |  |  | CHOP_44 |
|  |  |  | CHOP_45 |
|  | rs1794265 | upstream gene variant | CHOP_26 |
|  |  |  | CHOP_43 |
|  |  |  | CHOP_52 |
|  |  |  | CHOP_66 |
|  |  |  | CHOP_15 |
|  | rs4711279 | 5' UTR variant | CHOP_12 |
|  |  |  | CHOP_14 |
|  |  |  | CHOP_28 |
| Immunoglobulin A | rs9271366 | intergenic variant | CHOP_26 |
|  |  |  | CHOP_43 |
|  |  |  | CHOP_52 |
|  |  |  | CHOP_66 |
| Interstitial lung disease | rs3132946 | intron variant | CHOP_46 |
| Laryngeal squamous cell carcinoma | rs2857595 | intergenic variant | CHOP_2 |
|  |  |  | CHOP_32 |
|  |  |  | CHOP_33 |
|  |  |  | CHOP_39 |
| Late-onset myasthenia gravis | rs2071591 | intron variant | CHOP_2 |
|  | rs2071591 | intron variant | CHOP_33 |
| LDL cholesterol | rs3177928 | 3' UTR variant | CHOP_43 |
| Lumiracoxib-related liver injury | rs3129900 | intron variant | CHOP_26 |
|  |  |  | CHOP_43 |
|  |  |  | CHOP_52 |
|  |  |  | CHOP_66 |
| Lung adenocarcinoma | rs3117582 | intron variant | CHOP_12 |
|  |  |  | CHOP_14 |
|  |  |  | CHOP_23 |
|  |  |  | CHOP_28 |
|  |  |  | CHOP_31 |
|  |  |  | CHOP_4 |
|  |  |  | CHOP_48 |
|  |  |  | CHOP_56 |
|  |  |  | CHOP_61 |
|  |  |  | CHOP_65 |
| Lung cancer | rs2395185 | intron variant | CHOP_66 |
|  | rs3117582 | intron variant | CHOP_12 |
|  |  |  | CHOP_14 |
|  |  |  | CHOP_23 |
|  |  |  | CHOP_28 |
|  |  |  | CHOP_31 |
|  |  |  | CHOP_4 |
|  |  |  | CHOP_48 |
|  |  |  | CHOP_56 |
|  |  |  | CHOP_61 |
|  |  |  | CHOP_65 |
| Lymphoma | rs9268853 | intron variant | CHOP_66 |
| Marginal zone lymphoma | rs2922994 | upstream gene variant | CHOP_2 |
|  |  |  | CHOP_39 |
| Menopause (age at onset) | rs1046089 | missense variant | CHOP_23 |
|  |  |  | CHOP_61 |
| Metabolic syndrome | rs3099844 | downstream gene variant | CHOP_2 |
|  |  |  | CHOP_39 |
| Multiple sclerosis | rs3129889 | downstream gene variant | CHOP_26 |
|  |  |  | CHOP_43 |
|  |  |  | CHOP_52 |
|  |  |  | CHOP_66 |
|  | rs3129934 | intron variant | CHOP_26 |
|  |  |  | CHOP_43 |
|  |  |  | CHOP_52 |
|  |  |  | CHOP_66 |
|  | rs3135388 | downstream gene variant | CHOP_26 |
|  |  |  | CHOP_43 |
|  |  |  | CHOP_52 |
|  |  |  | CHOP_66 |
|  | rs9271366 | intergenic variant | CHOP_26 |
|  |  |  | CHOP_43 |
|  |  |  | CHOP_52 |
|  |  |  | CHOP_66 |
| Myasthenia gravis | rs9270986 | intron variant | CHOP_26 |
|  |  |  | CHOP_43 |
|  |  |  | CHOP_52 |
|  |  |  | CHOP_66 |
| Myositis | rs3130614 | intron variant | CHOP_2 |
|  |  |  | CHOP_39 |
| Neonatal lupus | rs3099844 | downstream gene variant | CHOP_2 |
|  |  |  | CHOP_39 |
| Parental longevity (mothers age at death) | rs1634726 | intron variant | CHOP_19 |
|  |  |  | CHOP_25 |
|  |  |  | CHOP_35 |
|  |  |  | CHOP_42 |
|  |  |  | CHOP_47 |
|  |  |  | CHOP_57 |
|  |  |  | CHOP_9 |

| Percentage gas trapping | rs2070600 | missense variant | CHOP_11 |
| --- | --- | --- | --- |
|  |  |  | CHOP_12 |
|  |  |  | CHOP_14 |
|  |  |  | CHOP_27 |
|  |  |  | CHOP_28 |
|  |  |  | CHOP_46 |
|  |  |  | CHOP_56 |
|  |  |  | CHOP_6 |
|  |  |  | CHOP_60 |
|  |  |  | CHOP_64 |
|  |  |  | CHOP_65 |
|  |  |  | CHOP_7 |
| Phospholipid levels (plasma) | rs3117181 | intron variant | CHOP_11 |
|  |  |  | CHOP_64 |
| Psoriasis | rs3134792 | intergenic variant | CHOP_2 |
|  |  |  | CHOP_39 |
| Pulmonary function | rs2070600 | missense variant | CHOP_11 |
|  |  |  | CHOP_12 |
|  |  |  | CHOP_14 |
|  |  |  | CHOP_27 |
|  |  |  | CHOP_28 |
|  |  |  | CHOP_46 |
|  |  |  | CHOP_56 |
|  |  |  | CHOP_6 |
|  |  |  | CHOP_60 |
|  |  |  | CHOP_64 |
|  |  |  | CHOP_65 |
|  |  |  | CHOP_7 |
|  | rs2857595 | intergenic variant | CHOP_2 |
|  |  |  | CHOP_32 |
|  |  |  | CHOP_33 |
|  |  |  | CHOP_39 |
| Response to angiotensin II receptor blocker therapy | rs7772131 | intron variant | CHOP_19 |
|  |  |  | CHOP_25 |
|  |  |  | CHOP_42 |
|  |  |  | CHOP_47 |
|  |  |  | CHOP_9 |
| Response to antipsychotic treatment | rs12526186 | intron variant | CHOP_42 |
|  |  |  | CHOP_9 |
| Rheumatoid arthritis | rs12194148 | upstream gene variant | CHOP_66 |
|  | rs12525220 | upstream gene variant | CHOP_26 |
|  |  |  | CHOP_43 |
|  |  |  | CHOP_66 |
|  | rs2157337 | upstream gene variant | CHOP_66 |
|  | rs6910071 | intron variant | CHOP_52 |
|  | rs805297 | intron variant | CHOP_23 |
|  |  |  | CHOP_61 |
| Rheumatoid arthritis (ACPA-negative) | rs2596565 | upstream gene variant | CHOP_2 |
|  |  |  | CHOP_39 |
| Schizophrenia | rs1046089 | missense variant | CHOP_23 |
|  |  |  | CHOP_61 |
|  | rs3131296 | intron variant | CHOP_11 |
|  |  |  | CHOP_27 |
|  |  |  | CHOP_46 |
|  |  |  | CHOP_52 |
|  |  |  | CHOP_60 |
|  |  |  | CHOP_64 |
|  |  |  | CHOP_7 |
| Stevens-Johnson syndrome and toxic epidermal necrolysis (SJS-TEN) | rs2734583 | intron variant | CHOP_2 |
|  |  |  | CHOP_39 |

| Systemic lupus erythematosus | rs1150753 | intron variant | CHOP_11 |
| --- | --- | --- | --- |
|  |  |  | CHOP_27 |
|  |  |  | CHOP_46 |
|  |  |  | CHOP_52 |
|  |  |  | CHOP_60 |
|  |  |  | CHOP_64 |
|  |  |  | CHOP_7 |
|  | rs1150754 | intron variant | CHOP_27 |
|  |  |  | CHOP_60 |
|  |  |  | CHOP_7 |
|  | rs1270942 | non coding transcript exon variant | CHOP_11 |
|  |  |  | CHOP_12 |
|  |  |  | CHOP_14 |
|  |  |  | CHOP_27 |
|  |  |  | CHOP_28 |
|  |  |  | CHOP_46 |
|  |  |  | CHOP_56 |
|  |  |  | CHOP_6 |
|  |  |  | CHOP_60 |
|  |  |  | CHOP_64 |
|  |  |  | CHOP_7 |
|  | rs3131379 | intron variant | CHOP_12 |
|  |  |  | CHOP_14 |
|  |  |  | CHOP_23 |
|  |  |  | CHOP_28 |
|  |  |  | CHOP_31 |
|  |  |  | CHOP_4 |
|  |  |  | CHOP_48 |
|  |  |  | CHOP_56 |
|  |  |  | CHOP_6 |
|  |  |  | CHOP_61 |
|  |  |  | CHOP_65 |
|  | rs558702 | intron variant | CHOP_12 |
|  |  |  | CHOP_14 |
|  |  |  | CHOP_23 |
|  |  |  | CHOP_28 |
|  |  |  | CHOP_31 |
|  |  |  | CHOP_4 |
|  |  |  | CHOP_48 |
|  |  |  | CHOP_56 |
|  |  |  | CHOP_6 |
|  |  |  | CHOP_61 |
|  |  |  | CHOP_65 |
|  | rs9267531 | non coding transcript exon variant | CHOP_12 |
|  |  |  | CHOP_14 |
|  |  |  | CHOP_23 |
|  |  |  | CHOP_28 |
|  |  |  | CHOP_31 |
|  |  |  | CHOP_4 |
|  |  |  | CHOP_48 |
|  |  |  | CHOP_56 |
|  |  |  | CHOP_61 |
|  |  |  | CHOP_65 |
| Systolic blood pressure | rs805303 | intron variant | CHOP_23 |
|  |  |  | CHOP_61 |
| Type 1 diabetes | rs9268645 | intron variant | CHOP_43 |

| Type 1 diabetes and autoimmune thyroid diseases | rs1270942 | non coding transcript exon variant | CHOP_11 |
| --- | --- | --- | --- |
|  |  |  | CHOP_12 |
|  |  |  | CHOP_14 |
|  |  |  | CHOP_27 |
|  |  |  | CHOP_28 |
|  |  |  | CHOP_46 |
|  |  |  | CHOP_56 |
|  |  |  | CHOP_6 |
|  |  |  | CHOP_60 |
|  |  |  | CHOP_64 |
|  |  |  | CHOP_7 |
|  | rs2523989 | missense variant | CHOP_13 |
|  | rs2857595 | intergenic variant | CHOP_2 |
|  |  |  | CHOP_32 |
|  |  |  | CHOP_33 |
|  |  |  | CHOP_39 |
|  | rs886424 | non coding transcript exon variant | CHOP_42 |
|  |  |  | CHOP_47 |
|  |  |  | CHOP_9 |
| Ulcerative colitis | rs2395185 | intron variant | CHOP_66 |
|  | rs9268853 | intron variant | CHOP_66 |
|  | rs9271366 | intergenic variant | CHOP_26 |
|  |  |  | CHOP_43 |
|  |  |  | CHOP_52 |
|  |  |  | CHOP_66 |
| Ulcerative colitis or Crohns disease | rs9271366 | intergenic variant | CHOP_26 |
|  |  |  | CHOP_43 |
|  |  |  | CHOP_52 |
|  |  |  | CHOP_66 |
| Visceral fat | rs13196329 | intron variant | CHOP_26 |
|  |  |  | CHOP_43 |
|  |  |  | CHOP_66 |
| Vitiligo | rs3823355 | upstream gene variant | CHOP_34 |
| Waist-to-hip ratio adjusted for body mass index | rs5020946 | downstream gene variant | CHOP_66 |

**Supplemental Table 3.** Each novel miRNA (89) that is in LD with a disease associated SNP as annotated by GWAS Catalog is reported along with the associated disease, SNP ID and genomic context of each variant (as annotated by GWAS catalog).

**
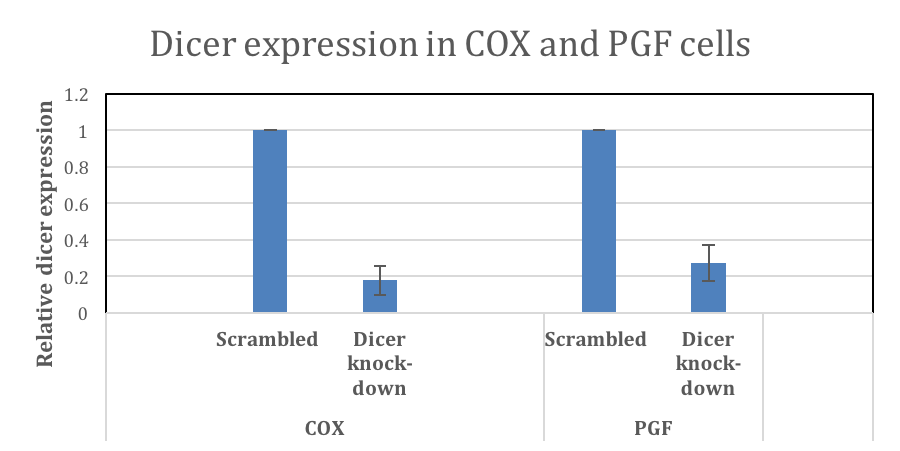
**

**Supplemental Figure 1.** Dicer was knocked-down in COX and PGF cells as described in materials and methods. Dicer expression was analyzed using q-PCR. Dicer expression in dicer knock-down cells is in reference to scrambled control set at 1. Dicer knock-down was tested 50 hours after first transduction. Cells were transduced for a total of 3 times as described in Methods. Cox dicer Scrambled Vs. knock-down T-test p=0.000222. PGF dicer scrambled vs. knock-down T-test p=0.001134
